# Effects of a Plastic-Free Lifestyle on Urinary Bisphenol A Levels in School-Aged Children of Southern Italy: A Pilot Study

**DOI:** 10.3389/fpubh.2021.626070

**Published:** 2021-02-01

**Authors:** Francesco Sessa, Rita Polito, Vincenzo Monda, Alessia Scarinci, Monica Salerno, Marco Carotenuto, Giuseppe Cibelli, Anna Valenzano, Angelo Campanozzi, Maria Pina Mollica, Marcellino Monda, Giovanni Messina

**Affiliations:** ^1^Department of Clinical and Experimental Medicine, University of Foggia, Foggia, Italy; ^2^Department of Advanced Medical and Surgical Sciences, University of Campania “Luigi Vanvitelli,” Naples, Italy; ^3^Section of Human Physiology and Unit of Dietetics and Sports Medicine, Department of Experimental Medicine, Università degli Studi della Campania “Luigi Vanvitelli,” Naples, Italy; ^4^Department of Education Sciences, Psychology, and Communication, University of Bari, Bari, Italy; ^5^Department of Medical, Surgical and Advanced Technologies “G.F. Ingrassia,” University of Catania, Catania, Italy; ^6^Clinic of Child and Adolescent Neuropsychiatry, Department of Mental Health, Physical and Preventive Medicine, Università degli Studi della Campania “Luigi Vanvitelli,” Naples, Italy; ^7^Pediatrics, Department of Medical and Surgical Sciences, University of Foggia, Foggia, Italy

**Keywords:** bisphenol A (BPA), urinary BPA concentration, pediatric population, eating behavior, endocrine disruptors (EDs), plastic food packaging (PFP)

## Abstract

Bisphenol A (BPA) is an endocrine disruptor (ED) frequently used in food packaging. BPA is used as a monomer in the manufacture of some food packaging. This study aimed to evaluate the urinary BPA concentration in an Italian pediatric cohort, testing the levels of this ED over a period of 6 months, evaluating the effects of a diet regimen with a reduction of Plastic Food Packaging (PFP). One hundred thirty Italian children were enrolled and divided into two groups “School Canteen” and “No School Canteen.” The first group consumed one meal at school using a plastic-free service for 5 days/weeks, while the other group did not modify their normal meal-time habits. The BPA levels were tested in urine samples at three time points: T0, is the time before the application of the plastic-free regimen diet; T3, 3 months later; and T6, 6 months later. A reduction of urine BPA levels was detected in the “School Canteen” group. In particular, the reduction was significant analyzing both the intra (among the three testing times) group and inter (between “School Canteen” and “No School Canteen”) group variability. Our results show the effects of a diet regimen with a reduction of PFP, demonstrating a connection between urinary BPA levels and food packaging.

## Introduction

Over the last few years, a new health concept has led us to consider a person's well-being in a broader way than in the past (1). Human wellness is conditioned by different essential factors, altering its homeostasis. Indeed, safety and human survival are strictly linked to environmental conditions. Many toxic substances act on human health, generating adverse effects. In this regard, the protracted exposure to minimal chemical substances can progressively alter the functioning of cells, tissues, and organs, as well as interfering with DNA expression (2).

Plastic Food Packaging (PFP) can interact with the food, generating the subsequent diffusion process of chemical compounds that could be transferred from packaging to food (3). This inconvenience is strictly related to different conditions such as the chemical properties of the PFP, storage temperature, UV exposure and the shelf life of the product. All these characteristics can determine the amount of the chemical substances that could be transferred: this process is named “migration” (4–6).

There is a growing interest in the possible health threat posed by chemical compounds of PFP known as endocrine disruptors (EDs). EDs are a heterogeneous group of substances that interfere with the endocrine system function (acting on the enzymes involved in steroidogenesis or interacting with the binding sites of sex hormones). They frequently act on steroid and thyroid homeostasis, generating several adverse outcomes such as behavioral disorders, obesity, some types of cancer (testicle, breast) and different problems of the reproductive systems (infertility, abortion, endometriosis). For these reasons, fetus development and childhood are considered the most susceptible periods (7).

Bisphenol A (BPA) is used as a monomer in the manufacture of some food packaging (such as polycarbonates) because it is regulated and has been approved to be used in the European union (EU) (8). Considering its characteristics, such as transparency, thermal and mechanical properties, it is also contained in epoxy resins (9, 10). Human exposure occurs through multiple sources, for example, drinking the water contained in plastic bottles, eating food contained in plastic packaging or food cans, and using dental devices (11).

To date, even if the safety of BPA is controversial, it can be used in the manufacture of PFP following the EU regulation on plastic material (8). Indeed, it is still not clear that BPA coming from food contact articles (very low levels) may pose a food safety issue. Several papers described that BPA may have estrogenic effects and may alter the thyroid function and the reproductive, nervous, and immune systems (12–14). Concerning the adverse effects on thyroid function, Andrianou et al. reported a positive association of BPA with thyroid-stimulating hormone (TSH), even if further studies are needed to confirm these interesting findings (15). Moreover, a recent post-mortem study performed on obese subjects showed the presence of BPA in two distinct regions of the human brain, suggesting that it may be able to cross the blood-brain barrier (16). Its adverse effects could be very important during puberty, particularly in females (17, 18). Furthermore, BPA levels could be linked to the development of different kinds of cancers (19–22). Moreover, several papers have described a direct link between BPA levels and increased risks of cardiovascular diseases and diabetes (23, 24): a recent study described an involvement of BPA in the development of both obesity and insulin resistance (25–27). Finally, several papers reported a pivotal role for BPA in obesogenic activity: it is able to act on adipocytes, reducing both the production and the secretion of adiponectin (28–32). Moreover, it increases the expression of the genes involved both in pro-inflammatory cytokine production and in lipid accumulation (30, 33). A recent review of Andra et al. suggested performing research on BPA and its analogs in order to better clarify the effects on human health (34).

This study aimed to evaluate the urinary BPA concentration in an Italian pediatric cohort, testing the levels of this ED over a 6-months period, evaluating the effects of a diet regimen with a reduction of food in plastic packaging. Moreover, the relationships among BPA, BMI, and plastic packaging use were investigated.

## Materials and Methods

### Participants

**Figure 1:**
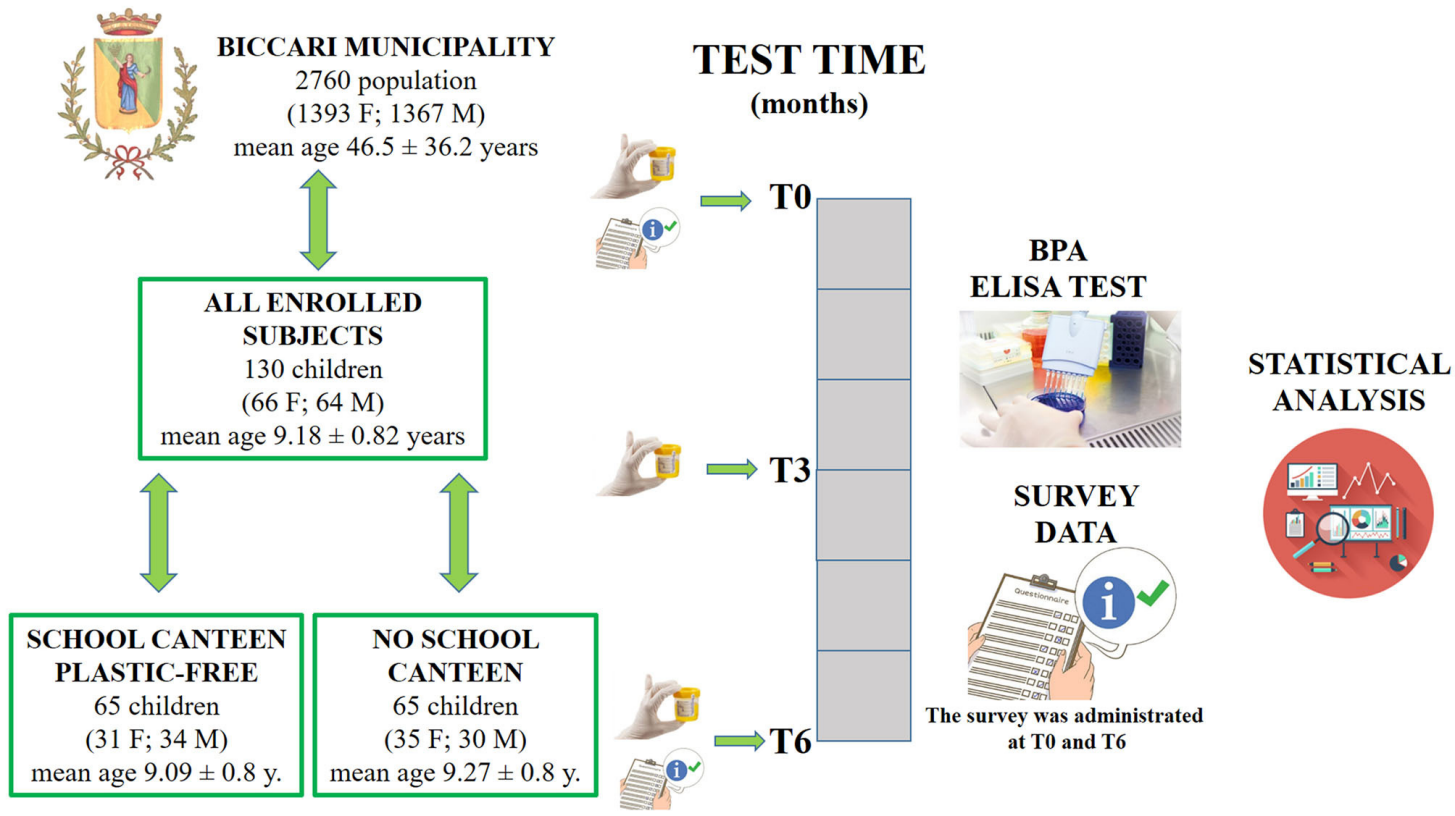
Three urine samples were collected at T0, T3, and T6. At T0 and T6 a questionnaire was filled in by the parents/guardians of each participant. At the end of the study, 390 urine samples had been analyzed.

One hundred and thirty Italian children were enrolled for this research study. Researchers provided the children's parents/guardians with information explaining the proposed research project and gave them the opportunity to opt their children out by filling in and returning a form. Written informed consent for publication was obtained from the parents/guardians of all the children enrolled. This research project was approved by the Ethics Committee of “Riuniti” Hospital (code: 28_09_2018_RH) and all procedures were performed in accordance with the Declaration of Helsinki. Children were divided into the “School Canteen” Group (65 children) and the “No School Canteen” Group (65 Children), and enrolled in September 2018. The main characteristics and the experimental model are summarized in Figure 1.

### Study Protocol

The study was performed in a small town in southern Italy (Biccari, Foggia, Apulia region, Italy). Before the school year, based on their own necessity, the parents/guardians of the children were free to choose a different diet regimen for their children. For this reason, the enrolled children were divided into two groups “School Canteen” and “No School Canteen”; the anthropometric data of the study population is summarized in Table 1; it is important to note that BMI was calculated using the child's weight and height and was then used to find the corresponding BMI-for-age percentile for the child's age and sex based on CDC growth charts for children and teens ages 2 through 19 years old (available at https://www.cdc.gov/healthyweight/bmi/calculator.html).

**Table 1:** Anthropometric data of study population.

	**School canteen group**	**No school canteen group**
Subjects	65 (31 F.; 34 M.)	65 (35 F.; 30 M.)
Mean age (y)	9.09 ± 0.8	9.27 ± 0.8
Mean height (cm)	137.7 ± 9.65	137.6 ± 8.76
Mean weight (Kg)	33.77 ± 8.33	33.46 ± 8.36
Mean BMI	17.64 ± 3.24	17.53 ± 3.48

The “School Canteen” group was made up of 65 children who ate 1 meal at school using a plastic-free service for 5 days/weeks: particularly, during school lunch they ate in the canteen, they used certified compostable materials, while in the previous school years, they had used all plastic material. The other group (65 children from the same school) did not modify their normal eating habits (e.g., using plastic bottles for water or another liquid, plastic plates, etc.). All parents/guardians of both groups declared that they avoided the usage of a microwave oven with plastic material for all the study period. No differences were detected between groups evaluating numerous measures of self-reported socioeconomic position, assessed through interviews conducted in-person by trained interviewers (35). Based on a certified release form from the family medical doctor, all participants were healthy. Three sampling points were identified to collect urine samples: - T0, is the time before the application of the plastic-free regimen diet; T3, 3 months later; and T6 6 months later. The survey cocerning the food habits related to the use of food packaging was administrated at T0 and T6.

The experimental model is illustrated in Figure 1.

### Sample Collection

Urine samples were taken from each participant after an overnight fast. Urine samples were collected in a glass tube. After the urine volume was determined, 5 mL were stored at −80°C in glass vials and analyzed within 1 month.

### BPA Determination in Urine

Total urinary BPA concentrations were determined by enzyme-linked immunoassay (ELISA), using a commercial kit, according to the manufacturer's instructions (Human Bisphenol A (BPA) ELISA kit MBS269957). Briefly, we added 100 μl of the sample and different concentrations of human BPA standard samples to corresponding wells and incubated them at 37°C for 90 min. After washing, we added 100 ml of the biotinylated human BPA antibody liquid to each well and incubated at 37°C for 60 min. We then added 100 μl of enzyme-conjugate liquid to the incubator at 37°C for 30 min. Finally, we added 100 μl of the color reagent to each well for the chromogenic reaction, and we read the plate at 450 nm. The urinary BPA levels are expressed in ng/ml, as indicated by the manufacturer.

Moreover, urinary creatinine concentrations were measured using a Creatinine Urinary Detection Kit following the user manual (Life Technologies Corporation, Carlsbad, CA, USA). Finally, the creatinine-standardized values (individual BPA concentrations divided by creatinine) were evaluated. The concentrations of the standard curve were 200, 100, 50, 25, 12.5, 6.25, ad 3.12 ng/ml. The blank had a reading equal to a BPA concentration lower than 0.05 ng/.

### Questionnaire

A questionnaire was administered to the parents/guardians of the enrolled children with the aim of investigating the food habits related to the use of food packaging. This survey had been adopted for a previous Italian study (36).

Six questions were investigated: Use of products with plasticized packaging for breakfast; Use of products with plasticized packaging for lunch/dinner; Use of daily snacks with plasticized packaging; Use of water in plastic bottles; Use of carbonated drinks in plastic bottles; Use of juices in plastic packs. For each answer a score was attributed: 0 (never), 1 (sometimes), 2 (Always). Therefore, the maximum score was 12 (the subject always used plastic packaging) and the minimum score 0 (the subject never used plastic packaging).

The questionnaire was administrated at T0 and T6.

### Statistical Analysis

As suggested by Stacy et al. (37), Log_10_-transformed urinary BPA concentrations were the outcome in all statistical analyses. Data were analyzed with the software package SPSS 22.0 for Windows. The two-way variance analysis (ANOVA) was used to determine any statistically significant differences among the groups. If needed, Tukey *post-hoc* test was used to calculate multiple comparisons.

## Results

**Figure 2:**
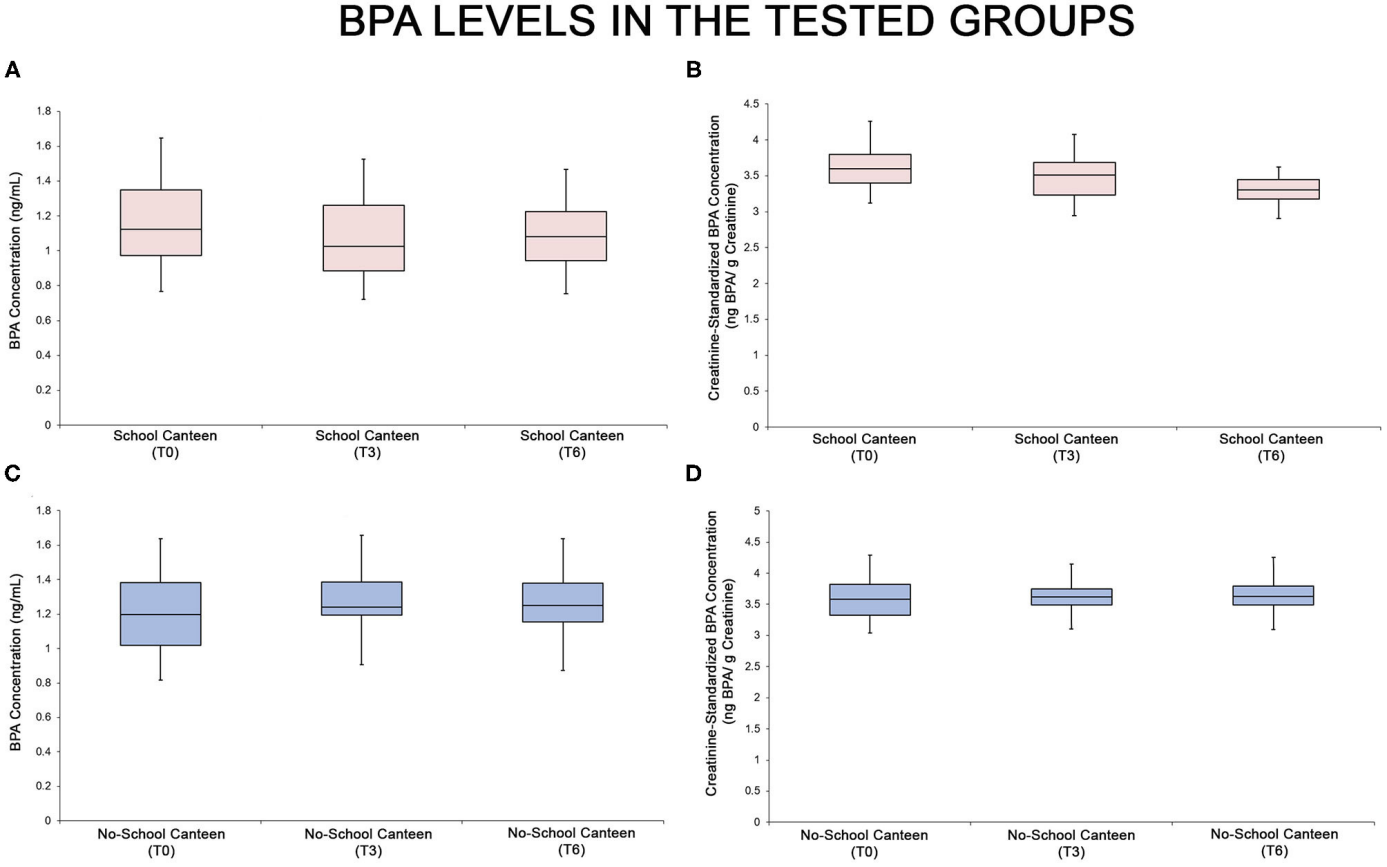
BPA levels in the two tested groups, “School Canteen” (**A,B**, pink box plots) and “No School Canteen” (**C,D**, blue box plots), at the different tested times. For each group, the BPA values are summarized in two different forms: unstandardized/unadjusted BPA values **(A,C)** and for creatinine-standardized BPA values **(B,D)**.

As summarized in Supplementary Tables 1–3 and in the relative box plot (Figure 2), a reduction of urinary BPA levels was detected after 6 months in the “School Canteen” group: the ANOVA test showed significant differences both for unstandardized/unadjusted BPA values [*F* = 3.066, *p* < 0.05, 0.00032, Figure 2A] and for creatinine-standardized BPA values [*F* = 3.066, *p* < 0.05, 5.93 × 10^−17^, Figure 2B]. No statistical differences were reported in the BPA values in the “No School Canteen” group (Figures 2C,D; Supplementary Table 1). The BPA values are summarized in two different forms: unstandardized/unadjusted BPA values (Figures 2A,C) and for creatinine-standardized BPA values (Figures 2B,D). It is interesting to note that analyzing the intra-group variability in the “School Canteen” group, the *post hoc* analysis highlighted significant differences analyzing the unstandardized/unadjusted BPA values between T0 and T6, while no significant differences were found at T0 vs. T3, and between T3 and T6 values (Supplementary Table 1). Moreover, as reported in Supplementary Tables 2, 3, the *post hoc* analysis ascertained that there were no significant differences between the two tested groups at T0 (*p* > 0.05), this became significant at T3 (*p* < 0.05) and T6 (*p* < 0.05) both for unstandardized/unadjusted BPA values and for creatinine-standardized BPA values.

The “School Canteen” group was subdivided under the BMI-for-age percentile criteria (using CDC growth charts for children and teens aged two through 19 years old into four groups: lower (13/65, less than the 5th percentile), normal (30/65, 5th percentile up to the 85th percentile), overweight (14/65, 85th to less than the 95th percentile) and obese (8/65, equal to or greater than the 95th percentile) (Table 2).

**Table 2:** The “School Canteen” group was subdivided under BMI criteria: the average BMI ± SD is summarized in the table.

**“Lower weigh” (BMI ± SD)**		**“Normal weight” (BMI ± SD)**		**“Overweight” (BMI ± SD)**		**“Obese” (BMI ± SD)**	
**13.16 ± 1.11**		**16.59 ± 1.32**		**19.65 ± 0.92**		**22.63 ± 1.89**	
**Male**	**Female**	**Male**	**Female**	**Male**	**Female**	**Male**	**Female**
13.48 ± 1.13	12.35 ± 0.67	16.47 ± 1.24	16.65 ± 1.39	19.22 ± 0.93	20.22 ± 0.64	23.22 ± 2.3	21.9 ± 1.08

**Figure 3:**
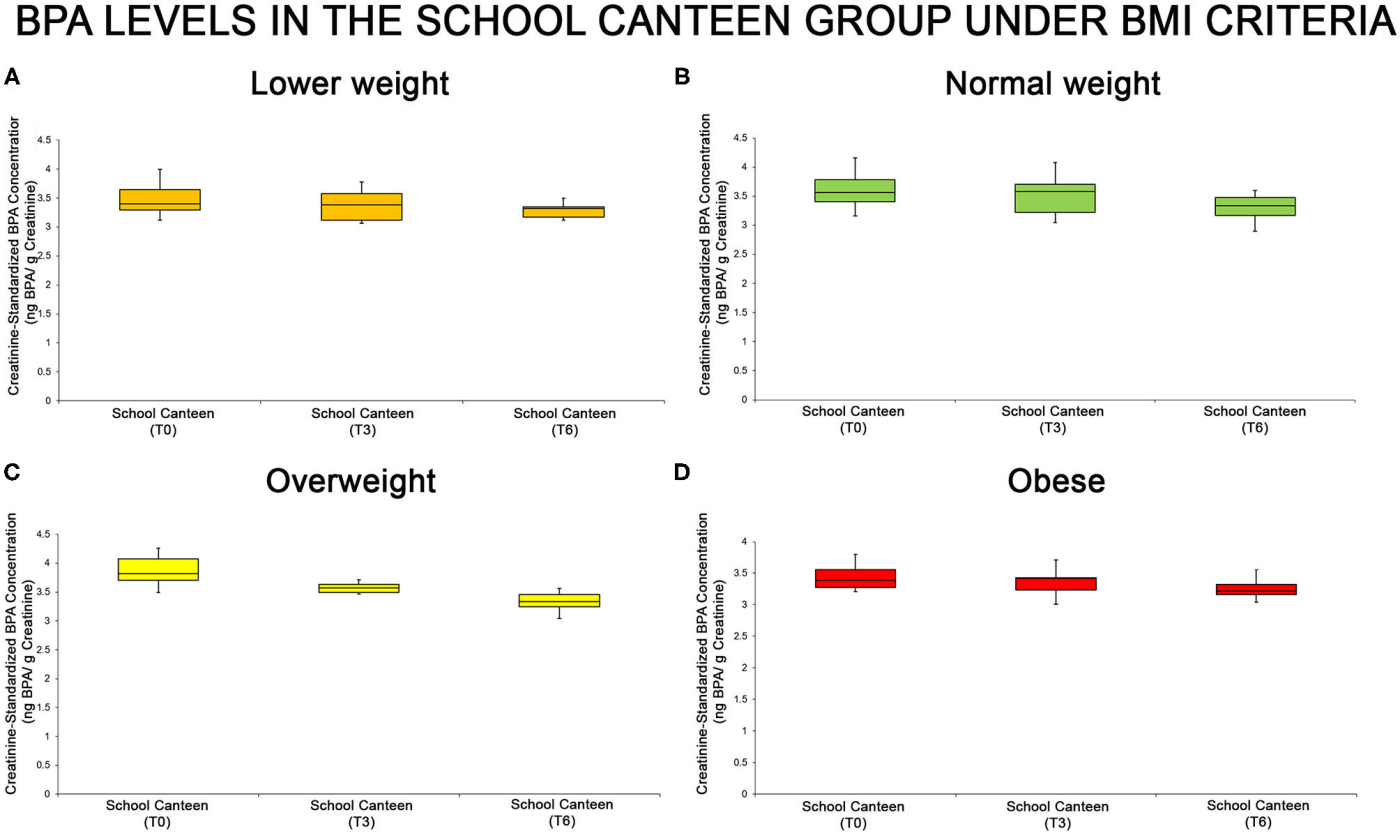
BPA levels at the different tested times in the “School Canteen” group, summarized under the BMI-for-age percentile criteria [lower weight, **(A)**, normal weight, **(B)**, overweight, **(C)**, obese, **(D)**]: even if the box plot analysis shows a reduction of creatinine-standardized BPA values in all sub-groups, significant differences were found in two of the four categories analyzed (normal weight and overweight).

The urine BPA levels of each sub-group are summarized through the box plot analysis (Figure 3): even if the box plot analysis shows a reduction of creatinine-standardized BPA values in all sub-groups, significant differences were found in two of the four categories analyzed (normal weight and overweight).

Moreover, the statistical analysis was performed analyzing the intra group variation. In the “Normal weight” and “Overweight” groups there were significant differences in the creatinine-standardized BPA levels as reported in Supplementary Table 4. As summarized in Supplementary Table 5, in the “Normal weight” group there are significant differences at T0 compared with T6, and at T3 compared with T6 (*P* < 0.05). No statistical differences were reported comparing T0 with T3 values. In the “Overweight” group, there were significant differences among all sampling points T0 vs. T3, T3 vs. T6, T0 vs. T6 (*P* > 0.05). In the “Lower weight” and “Obese” groups no significant differences were reported (T0 vs. T3, *p* > 0.05; T3 vs. T6, *p* > 0.05; T0 vs. T6, *p* > 0.05).

The same subdivision (BMI-for-age percentile criteria) was performed in the “No-School Canteen” group, generating four groups: lower (15/65), normal (26/65), overweight (14/65) and obese (10/65) (Table 3).

**Table 3:** The “No-School Canteen” group was subdivided under BMI criteria: the average BMI ± SD is summarized in the table.

**“Lower weigh” (BMI ± SD)**		**“Normal weight” (BMI ± SD)**		**“Overweight” (BMI ± SD)**		**“Obese” (BMI ± SD)**	
**13.1 ± 0.86**		**16.78 ± 1.25**		**19.83 ± 1.03**		**22.93 ± 2.01**	
**Male**	**Female**	**Male**	**Female**	**Male**	**Female**	**Male**	**Female**
13.6 ± 1.05	12.76 ± 0.54	16.58 ± 1.18	16.95 ± 1.33	18.97 ± 0.82	20.49 ± 0.62	23.62 ± 2.27	21.9 ± 1.08

Moreover, in the same group the statistical analysis was performed evaluating the BPA levels; nevertheless, no significant differences were observed (Supplementary Table 7).

**Figure 4:**
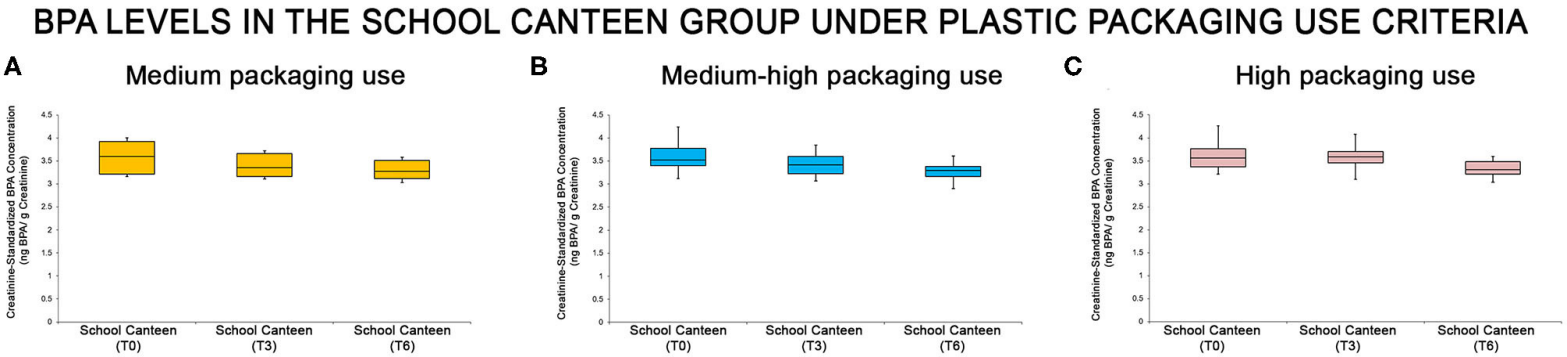
BPA levels at the different tested times in the “School Canteen” group, summarized under the plastic packaging use criteria [medium packaging use, **(A)**, medium-high packaging use, **(B)**, high packaging use, **(C)**]: even if the box plot analysis showed a reduction of creatinine-standardized BPA values in all sub-groups, significant differences were found in two of the three categories analyzed (medium-high packaging use and high packaging use).

The “School Canteen” group was subdivided under packaging use criteria, analyzing the data of the survey in four groups: Score 0–3 (low plastic packaging use, 0/65); score 4–6 (medium plastic packaging use, 8/65); score 7–9 (medium-high plastic packaging use, 39/65); score 10–12 (high plastic packaging use, 18/65). As reported, only 12.3% of this group could be classified as having a virtuous behavior regarding the use of plastic packaging. The urine BPA levels of each sub-group were summarized through the box plot analysis (Figure 4): even if the box plot analysis showed a reduction of creatinine-standardized BPA values in all sub-groups, significant differences were found in two of the three categories analyzed (medium-high packaging use and high packaging use).

Statistical analysis was performed analyzing the intra group variation. In the “Medium-high packaging use” and “high packaging use” groups there were significant differences in the creatinine-standardized BPA levels, as reported in Supplementary Table 6. As summarized in Supplementary Table 7, in the “Medium-high packaging use” group there are significant differences at T0 compared with T6, and at T3 compared with T6 (*P* < 0.05). No statistical differences were reported comparing T0 with T3 values. In the “high packaging use” group, there are significant differences among all sampling points T0 vs. T3, T3 vs. T6, T0 vs. T6 (*P* > 0.05). In the “Medium packaging use” group no significant differences were reported (T0 vs. T3, *p* > 0.05; T3 vs. T6, *p* > 0.05; T0 vs. T6, *p* > 0.05). It is important to note that the number of subjects in this subgroup (8/65) is limited.

## Discussion

The international data concerning the transfer between packaging and the food it contains are constantly growing. The so-called “migration” process is related to different parameters such as the nature of the food, the concentration of substances in the packaging composition, storage temperature, and contact duration (38–40). The adverse effects of these substances affect the reproductive systems, exerting (41) their action particularly during puberty (42, 43). In several countries, such as Italy, even if the public water supply is potable, a lot of people drink from plastic bottles rather than drinking tap water (44). Plastic bottles usually contain a higher concentration of several chemical compounds that could be transferred to the contained liquid such as water, alcoholic and nonalcoholic beverages (45). These chemical compounds are released under different conditions such as high temperatures or UV exposure; the owners of storage facilities for bottled water often disregard warnings, storing the bottles in open spaces under these adverse environmental conditions (46–48). This concept is shown by Makris et al. in their experimental model where they showed that high urinary BPA levels were partially ascribed to the high polycarbonate water consumption in the summer and weather characteristics (high temperatures, >40°C; very high UV index values, >8), which could be causing BPA leaching from polycarbonate (41). The presence of BPA in drinking water is controversial, even if it is certainly released by polycarbonate food packaging (49).

In light of this evidence, in this study, we evaluated, for the first time, the urinary BPA concentration in an Italian pediatric cohort over 6 months, assessing the effects of a diet regimen with a reduction of PFP. Our results show a reduction of urine BPA levels after 6 months in the “School Canteen” group: the reduction is significant analyzing both the intra (among the tested periods, T0, T3, and T6) group and inter (between “School Canteen” and “No School Canteen”) group variability. We found that there were significant differences in urinary BPA concentrations in the “School Canteen” group after 6 months compared to T0 and the BPA reduction correlated with the score of the group, which could be classified as having a virtuous behavior compared to the use of plastic packaging. These findings are very interesting, highlighting how lifestyle habits can change and improve our health status.

The “School Canteen” group was subdivided under the BMI criteria, generating lower, normal, overweight, and obese groups. The results show that the reduction of urinary BPA values was detected in children in two categories (normal and overweight), while no statistically significant differences were detected in the lower and obese groups. In the lower weight group this result was related to the degree of BMI: indeed, in consideration of their diet habit, it could be supposed that a single correction in diet regimen should not influence the BPA levels. Moreover, in the obese subjects BPA remained about the same: for this group, a correction in the diet regimen did not significantly influence the BPA urinary levels. It is important to remark that the data about the “obese” group could be influenced by the small number of subjects in this group. Fernandez et al. also reported a non-significant negative association between BMI and adipose BPA concentration, which suggests that even if BPA is stored in adipose tissue, a higher BMI may not predict elevated BPA biomarker concentrations (50). Previous studies reported that perinatal exposure to BPA can predispose both to obesity and insulin resistance, particularly in older age (51–53). Furthermore, in pediatric populations higher levels of BPA are strictly associated with obesity and insulin resistance (54). Several recent epidemiological studies have demonstrated a correlation between urinary BPA levels and obesity (55, 56). Moreover, as previously described, the exposure to BPA can induce adverse effects on neurological control, suggesting an involvement in behavior management (57–59). A very recent study performed on obese subjects demonstrated a reduction in most phthalate levels after a dietary intervention (60). The results of the present study are in agreement with these studies, even if this indication should be confirmed by further studies. Moreover, analyzing the survey results, the urinary BPA levels of the “School Canteen” group was further subdivided under packaging use criteria. In particular, for each test time, 4 subgroups were obtained: low plastic packaging use group; medium plastic packaging use group; medium-high plastic packaging use group; and high plastic packaging use group. The urinary BPA levels were significantly reduced at the three sampling points in the groups with medium-high and high plastic packaging use. No significant differences at the three sampling points were found in the other groups, even if it is important to remark that the results of “Medium packaging use” group could be influenced by the small number of subjects. These results suggest that a few changes in the use of plastic packaging can influence the urinary BPA levels in the children that had intensively used plastic packaging, while no significant changes were found in the “Medium packaging use” group. In the same way, children who rarely consumed food in plastic packaging showed no significant reduction of their BPA levels. These data suggest that BPA urinary levels are strictly related to PFP use. As reported by Heras-Gonzales et al., there is a strong correlation between obesity, lifestyle and diet and exposure to ED chemicals. Several studies reported that the migration of BPA from drink and food packaging, plastic baby bottles, and the coating of cans is associated with obesity, classifying them as obesogens (61). Diet also strongly influences urinary BPA levels and changes in diet are attributable to alterations in urinary BPA levels: the results of the present study suggest that a plastic-free lifestyle may be related to a reduction in urinary BPA levels. In fact, as reported by Carnile and Michels, the vast majority of BPA exposure occurs through the ingestion of contaminated food and drink and metabolization is rapid and complete (55). The dosage of BPA urinary levels represents the prevalent way to determine BPA levels in the human body, not only because it potentially reaches more people without age differentiation (children and adults), but also because it inadvertently occurs over long time periods, almost certainly over one's entire life (62). The data obtained in the present study are in agreement with previous published papers. Nevertheless, it is important to note that these considerations are based on the questionnaire results: this represents a limit for the study, because the answers provided by parents/guardians may not completely reflect the child's BPA exposure. In light of these results, it may be desirable that several countermeasures should be taken in order to develop effective interventions that are feasible in the general population. In this way, each action should be carefully evaluated because it could be ineffective (63). At this regard, Rudel et al. reported that a dietary intervention of 3 days of eating food with limited food packaging was associated with substantial reductions in BPA exposure (64). The statistical reduction of BPA concentration that we found in our pediatric population confirmed these data, showing the effects of a diet regimen with a decrease of food in plastic packaging, demonstrating that there is a connection between urinary BPA levels and PFP. Many literature data also reported that urinary BPA concentrations are associated with behavior problems, suggesting that BPA may predominantly affect the behavior of children rather than their cognitive function (65, 66). In addition, several studies reported the obsogenic effects of BPA, suggesting that BPA may cause lipid accumulation and promote the differentiation of pre-adipose cells via the peroxisome proliferator-activated receptor-γ (PPARG) pathway (67). It has also been reported that BPA increased adipogenic markers in Murine 3T3-L1 preadipocytes. Moreover, in children, BPA levels were significantly correlated with insulin resistance, albuminuria, and irregular vascular function (68).

This study has several strengths: the urine samples were obtained in homogenous groups following both age and sociodemographic criteria. Indeed, several previous studies reported that children or adolescents also have decreased urinary BPA concentrations with increasing age (69–71). Other studies reported that sociodemographic factors such as race, education, and household income may influence childhood exposure to BPA (37, 72). Moreover, the BPA urinary levels were evaluated using the creatinine-standardized BPA concentrations (73).

It is important to note that this study has several limitations. The urinary BPA concentrations were tested through ELISA-test. Even if a series of highly sensitive ELISA tests in direct and indirect assay formats with high specificity has been developed (74), the high-performance liquid chromatography-tandem mass spectrometry (LC-MS/MS) method remains the best method (75); however, the ELISA-test represents a reliable, cheaper, and faster quantitative test; moreover, it is available in all laboratories and does not require specialized personnel. Moreover, the data about the use of plastic packaging were obtained through a questionnaire: this can be considered an inherent limitation because the answers provided by parents/guardians may not reflect the child's real exposure; furthermore, even if the same questionnaire had been adopted for a previous national study, the BPA exposure values might be influenced by the scoring system. Moreover, analyzing the data about the packaging use criteria the results of the “Medium packaging use” group could be influenced by the small number of the subjects I this group (8/65). Finally, the data about the BMI criteria discussed in the present study could be influenced by the small number of children in the “obese” group (8/65).

## Conclusions

The most recent studies, both *in vitro* and *in vivo*, demonstrate that human exposure to BPA is related to several adverse effects, particularly in the first period of life. It is commonly ingested involuntarily through diet, both drinking and eating substances packaged in plastic containers. Even if further research is needed, the results of the present study suggest that a substantial reduction in the use of PFP directly correlates with a decrease of urinary BPA concentration and an active lifestyle. The idea that increasing awareness of how health and human safety could be linked to environmental conditions and chemical compound exposure represents undoubtful an important concept to develop future studies and applications. Particularly, in the new concept to life transitioning toward a plastic-free world.

## Data Availability Statement

The datasets presented in this study can be found in online repositories. The names of the repository/repositories and accession number(s) can be found in the article/Supplementary Material.

## Ethics Statement

The studies involving human participants were reviewed and approved by Ethics Committee of Riuniti Hospital. Written informed consent to participate in this study was provided by the participants' legal guardian/next of kin.

## Author Contributions

FS and GM: conceptualization and project administration. FS, RP, VM, MM, and GM: methodology and resources. FS, RP, AS, MS, MC, GC, AV, MPM, and GM: software, formal analysis, and investigation. FS, RP, VM, AC, MM, and GM: validation. FS and RP: data curation. FS, RP, and GM: writing—original draft preparation and writing—review and editing. MM and GM: supervision. All authors have read and agreed to the published version of the manuscript.

## Conflict of Interest

The authors declare that the research was conducted in the absence of any commercial or financial relationships that could be construed as a potential conflict of interest.
