# Supplementary material for: Effects of a Plastic-Free Lifestyle on Urinary Bisphenol A Levels in School-Aged Children of Southern Italy: A Pilot Study
Source: Front Public Health. 2021 Feb 1;9:626070. doi: 10.3389/fpubh.2021.626070 (PMC7882684; doi:10.3389/fpubh.2021.626070)
Supplement: Supplementary file 1 [file Table_1.DOCX]

**SUPPLEMENTARY MATERIAL**

|  | **T0 vs T3** | **T3 vs T6** | **T0 vs T6** |
| --- | --- | --- | --- |
| **BPA Level**  **“School Canteen”** | *n.s.* | *n.s.* | **P<0.05*** |
| **BPA Level**  **“No School Canteen”** | *n.s.* | *n.s.* | *n.s.* |
| **Creatinine-Standardized BPA Level**  **“School Canteen”** | **P<0.05*** | **P<0.05*** | **P<0.05*** |
| **Creatinine-Standardized BPA Level**  **“No School Canteen”** | *n.s.* | *n.s.* | *n.s.* |

**Table S1.** P values of the post hoc analysis, evaluating intragroup variability.

| **Label** | **BPA average concentration**  **(ng/ml) ± SD** | **BPA minimum value**  **(ng/ml)** | **Quartile**  **1 value**  **(ng/ml)** | **BPA median value**  **(ng/ml)** | **Quartile**  **3 value**  **(ng/ml)** | **BPA maximum value**  **(ng/ml)** | **P value** |
| --- | --- | --- | --- | --- | --- | --- | --- |
| **“School Canteen”**  **(T0)** | 1.16 ± 0.24 | 0.76 | 0.97 | 1.12 | 1.34 | 1.64 | *n.s.* |
| **“No School Canteen”**  **(T0)** | 1.21 ± 0.24 | 0.81 | 1.01 | 1.19 | 1.38 | 1.63 |  |
| **“School Canteen”**  **(T3)** | 1.08 ± 0.22 | 0.72 | 0.88 | 1.02 | 1.26 | 1.52 | **<0.05** |
| **“No School Canteen”**  **(T3)** | 1.25 ± 0.17 | 0.88 | 1.19 | 1.24 | 1.38 | 1.65 |  |
| **“School Canteen”**  **(T6)** | 1.08 ± 0.18 | 0.75 | 0.94 | 1.08 | 1.22 | 1.46 | **<0.05** |
| **“No School Canteen”**  **(T6)** | 1.25 ± 0.17 | 0.87 | 1.15 | 1.25 | 1.38 | 1.63 |  |

**Table S2.** BPA levels at the different tested times: the post hoc analysis ascertained that there were no significant differences between the two tested groups at T0, this became significant at T3 and T6.

| **Label** | **Creatinine-Standardized BPA average concentration**  **(µg BPA/g Creatinine) ± SD** | **Creatinine-Standardized BPA minimum value**  **(µg BPA/g Creatinine)** | **Quartile**  **1 value**  **(µg BPA/g Creatinine)** | **Creatinine-Standardized BPA median value**  **(µg BPA/g Creatinine)** | **Quartile**  **3 value**  **(µg BPA/g Creatinine)** | **Creatinine-Standardized BPA maximum value**  **(µg BPA/g Creatinine)** | **P value** |
| --- | --- | --- | --- | --- | --- | --- | --- |
| **“School Canteen”**  **(T0)** | 3.62 ± 0.28 | 3.11 | 3.4 | 3.6 | 3.8 | 4.26 | *n.s.* |
| **“No School Canteen”**  **(T0)** | 3.59 ± 0.33 | 3.04 | 3.32 | 3.58 | 3.82 | 4.29 |  |
| **“School Canteen”**  **(T3)** | 3.47 ± 0.26 | 2.94 | 3.23 | 3.51 | 3.68 | 4.07 | **<0.05** |
| **“No School Canteen”**  **(T3)** | 3.61 ± 0.22 | 3.1 | 3.49 | 3.61 | 3.75 | 4.22 |  |
| **“School Canteen”**  **(T6)** | 3.31 ± 0.17 | 2.9 | 3.17 | 3.3 | 3.44 | 3.62 | **<0.05** |
| **“No School Canteen”**  **(T6)** | 3.63 ± 0.24 | 3.09 | 3.49 | 3.62 | 3.79 | 4.29 |  |

**Table S3.** BPA levels at the different tested times: the post hoc analysis ascertained that there were no significant differences between the two tested groups at T0, this became significant at T3 and T6.

| **“School Canteen” Group** | **Creatinine-Standardized BPA average concentration**  **(µg BPA/g Creatinine) ± SD** | **Creatinine-Standardized BPA minimum value**  **(µg BPA/g Creatinine)** | **Quartile**  **1 value**  **(µg BPA/g Creatinine)** | **Creatinine-Standardized BPA median value**  **(µg BPA/g Creatinine)** | **Quartile**  **3 value**  **(µg BPA/g Creatinine)** | **Creatinine-Standardized BPA maximum value**  **(µg BPA/g Creatinine)** | **P value** |
| --- | --- | --- | --- | --- | --- | --- | --- |
| **“Lower weight”**  **(T0)** | 3.48 ± 0.3 | 3.12 | 3.29 | 3.4 | 3.65 | 4 | *n.s.* |
| **“Lower weight”**  **(T3)** | 3.37 ± 0.28 | 3.07 | 3.11 | 3.38 | 3.58 | 3.78 |  |
| **“Lower weight”**  **(T6)** | 3.28 ± 0.13 | 3.12 | 3.17 | 3.32 | 3.34 | 3.5 |  |
| **“Normal weight”**  **(T0)** | 3.58 ± 0.27 | 3.16 | 3.4 | 3.56 | 3.78 | 4.16 | **<0.05** |
| **“Normal weight”**  **(T3)** | 3.48 ± 0.27 | 3.05 | 3.22 | 3.58 | 3.7 | 4.07 |  |
| **“Normal weight”**  **(T6)** | 3.3 ± 0.19 | 2.9 | 3.17 | 3.34 | 3.48 | 3.6 |  |
| **“Overweight”**  **(T0)** | 3.87 ± 0.28 | 3.49 | 3.7 | 3.82 | 4.07 | 4.26 | **<0.05** |
| **“Overweight”**  **(T3)** | 3.57 ± 0.09 | 3.47 | 3.49 | 3.57 | 3.63 | 3.71 |  |
| **“Overweight”**  **(T6)** | 3.33 ± 0.17 | 3.04 | 3.24 | 3.33 | 3.46 | 3.56 |  |
| **“Obese”**  **(T0)** | 3.42 ± 0.19 | 3.21 | 3.27 | 3.39 | 3.56 | 3.8 | *n.s.* |
| **“Obese”**  **(T3)** | 3.35 ± 0.21 | 3.01 | 3.23 | 3.42 | 3.43 | 3.71 |  |
| **“Obese”**  **(T6)** | 3.27 ± 0.19 | 3.04 | 3.16 | 3.22 | 3.32 | 3.61 |  |

**Table S4.** BPA levels at the different tested times in the “School Canteen” group, summarized under the BMI criteria: significant differences were found in the urinary BPA values in the children in three of the four categories analyzed (lower, normal and overweight).

|  | **T0 vs T3** | **T3 vs T6** | **T0 vs T6** |
| --- | --- | --- | --- |
| **Creatinine-Standardized BPA Level**  **“Lower weight”** | *n.s.* | *n.s.* | *n.s.* |
| **Creatinine-Standardized BPA Level**  **“Normal weight”** | *n.s.* | **P<0.05*** | **P<0.05*** |
| **Creatinine-Standardized BPA Level**  **“Overweight”** | **P<0.05*** | **P<0.05*** | **P<0.05*** |
| **Creatinine-Standardized BPA Level**  **“Obese”** | *n.s.* | *n.s.* | *n.s.* |

**Table S5.** P values of the post hoc analysis, evaluating intragroup variability.

| **“No School Canteen” Group** | **Creatinine-Standardized BPA average concentration**  **(µg BPA/g Creatinine) ± SD** | **Creatinine-Standardized BPA minimum value**  **(µg BPA/g Creatinine)** | **Quartile**  **1 value**  **(µg BPA/g Creatinine)** | **Creatinine-Standardized BPA median value**  **(µg BPA/g Creatinine)** | **Quartile**  **3 value**  **(µg BPA/g Creatinine)** | **Creatinine-Standardized BPA maximum value**  **(µg BPA/g Creatinine)** | **P value** |
| --- | --- | --- | --- | --- | --- | --- | --- |
| **“Lower weight”**  **(T0)** | 3.48 ± 0.26 | 3.04 | 3.3 | 3.49 | 3.65 | 4.06 | *n.s.* |
| **“Lower weight”**  **(T3)** | 3.61 ± 0.15 | 3.28 | 3.55 | 3.62 | 3.74 | 3.82 |  |
| **“Lower weight”**  **(T6)** | 3.62 ± 0.18 | 3.17 | 3.56 | 3.66 | 3.72 | 3.92 |  |
| **“Normal weight”**  **(T0)** | 3.51 ± 0.36 | 3.04 | 3.17 | 3.53 | 3.8 | 4.29 | *n.s* |
| **“Normal weight”**  **(T3)** | 3.52 ± 0.21 | 3.1 | 3.36 | 3.54 | 3.66 | 3.93 |  |
| **“Normal weight”**  **(T6)** | 3.57 ± 0.23 | 3.09 | 3.36 | 3.54 | 3.66 | 3.93 |  |
| **“Overweight”**  **(T0)** | 3.78 ± 0.31 | 3.24 | 3.51 | 3.87 | 4.02 | 4.27 | *n.s* |
| **“Overweight”**  **(T3)** | 3.77 ± 0.23 | 3.48 | 3.59 | 3.71 | 3.93 | 4.22 |  |
| **“Overweight”**  **(T6)** | 3.75 ± 0.3 | 3.2 | 3.51 | 3.79 | 3.9 | 3.29 |  |
| **“Obese”**  **(T0)** | 3.7 ± 0.28 | 3.17 | 3.58 | 3.74 | 3.81 | 4.26 | *n.s.* |
| **“Obese”**  **(T3)** | 3.62 ± 0.19 | 3.18 | 3.56 | 3.63 | 3.75 | 3.89 |  |
| **“Obese”**  **(T6)** | 3.64 ± 0.2 | 3.41 | 3.47 | 3.62 | 3.72 | 4.07 |  |

**Table S6.** BPA levels at the different tested times in the “No-School Canteen” group, summarized under the BMI criteria: no significant differences were found.

|  | **“School Canteen” Group under packaging use criteria** | **Creatinine-Standardized BPA average concentration**  **(µg BPA/g Creatinine) ± SD** | **Creatinine-Standardized BPA minimum value**  **(µg BPA/g Creatinine)** | **Quartile**  **1 value**  **(ng/ml)** | **Creatinine-Standardized BPA median value**  **(µg BPA/g Creatinine)** | **Quartile**  **3 value**  **(ng/ml)** | **Creatinine-Standardized BPA maximum value**  **(µg BPA/g Creatinine)** | **P value** |
| --- | --- | --- | --- | --- | --- | --- | --- | --- |
| **medium plastic packaging use** | **Score 4-6”**  **(T0)** | 3.58 ± 0.4 | 3.16 | 3.21 | 3.6 | 3.92 | 4 | *n.s.* |
|  | **Score 4-6”**  **(T3)** | 3.39 ± 0.28 | 3.11 | 3.16 | 3.35 | 3.66 | 3.72 |  |
|  | **Score 4-6”**  **(T6)** | 3.3 ± 0.23 | 3.03 | 3.12 | 3.27 | 3.51 | 3.58 |  |
| **medium-high plastic packaging use** | **Score 7-9”**  **(T0)** | 3.59 ± 0.29 | 3.11 | 3.4 | 3.52 | 3.77 | 4.24 | **<0.05** |
|  | **Score 7-9”**  **(T3)** | 3.42 ± 0.23 | 3.07 | 3.22 | 3.41 | 3.6 | 3.84 |  |
|  | **Score 7-9”**  **(T6)** | 3.28 ± 0.17 | 2.9 | 3.16 | 3.29 | 3.37 | 3.61 |  |
| **high plastic packaging use** | **Score 10-12”**  **(T0)** | 3.56 ± 0.28 | 3.21 | 3.37 | 3.56 | 3.76 | 4.26 | **<0.05** |
|  | **Score 10-12”**  **(T3)** | 3.42 ± 0.25 | 3.01 | 3.46 | 3.58 | 3.7 | 4.07 |  |
|  | **Score 10-12”**  **(T6)** | 3.34 ± 0.17 | 3.04 | 3.21 | 3.31 | 3.48 | 3.6 |  |

**Table S7.** BPA levels at the different tested times in the “School Canteen” group, summarized under the BMI criteria: significant differences were found in the urinary BPA values in the children in three of the four categories analyzed (medium, medium-high, high plastic packaging use).

|  | **T0 vs T3** | **T3 vs T6** | **T0 vs T6** |
| --- | --- | --- | --- |
| **Creatinine-Standardized BPA Level**  **“low plastic”** | *n.s.* | *n.s.* | *n.s.* |
| **Creatinine-Standardized BPA Level**  **“medium plastic”** | **P<0.05*** | **P<0.05*** | **P<0.05*** |
| **Creatinine-Standardized BPA Level**  **“high plastic”** | *n.s.* | **P<0.05*** | **P<0.05*** |

**Table S8.** P values of the post hoc analysis, evaluating intragroup variability.
